# Toll-Like Receptors in Angiogenesis

**DOI:** 10.1100/tsw.2011.92

**Published:** 2011-04-19

**Authors:** Karsten Grote, Harald Schütt, Bernhard Schieffer

**Affiliations:** Cardiology and Angiology, Hannover Medical School, Hannover, Germany

**Keywords:** Toll-like receptors, angiogenesis

## Abstract

Toll-like receptors (TLRs) are known as pattern-recognition receptors related to the Toll protein of *Drosophila*. After recognition of pathogen-associated molecular patterns of microbial origin, the TLRs alert the immune system, and initiate innate and adaptive immune responses. The TLR system, though, is not confined solely to the leukocyte-mediated immune defense against exogenous pathogens. Besides myeloid cells, TLR expression has been reported in multiple tissues and cell types, including epithelial and endothelial cells. Moreover, despite the microbial patterns that are commonly accepted as TLR ligands, there is increasing evidence that TLRs also recognize host-derived molecules. In this regard, recent studies point to an involvement of TLRs in various chronic inflammatory disorders and cardiovascular diseases, including atherosclerosis, rheumatoid arthritis, systemic lupus erythematosus, and even cancer. A common feature of these disorders is an enhanced so-called inflammation-induced angiogenesis. However, inflammation-induced angiogenesis is not solely a key component of pathogen defense during acute infection or chronic inflammatory disorders, but also plays a critical role in repair mechanisms, e.g., wound healing and subsequent tissue regeneration. Interestingly, the latest research could coincidentally demonstrate that TLR activation promotes angiogenesis in various inflammatory settings in response to both exogenous and endogenous ligands, although the precise mode of action of TLRs in this context still remains ambiguous. The objective of this review is to present evidence for the implication of TLRs in angiogenesis during physiological and pathophysiological processes, and the potential clinical relevance for new treatment regimes involving TLR modulation.

## TOLL-LIKE RECEPTORS – MORE THAN PATHOGEN RECOGNITION

Mammalian homologues of the prominent *Drosophila* Toll protein were discovered in the mid-1990s[1,2]. An early highlight in mammalian Toll-like receptor (TLR) discovery was the identification of TLR4 as the functional receptor for bacterial lipopolysaccharide (LPS) by Bruce Beutler[3]. Since then, the number of known TLRs has increased to comprise an entire receptor protein family[4], with 10 members in humans and even 11 members in mice so far. All of them have initially been discovered as sentinels of innate immunity, recognizing invading pathogens directly on the plasma membrane or after phagocytosis on the membrane of endosomes. In this regard, TLRs sense a diversity of pathogenassociated molecular patterns (PAMPs) of bacterial and viral origin in order to organize the body's immune defense (Fig. 1). This is accomplished by the activation of inflammatory pathways, such as the mitogen-activated protein kinase (MAPK) cascade or nuclear factor-κB (NF-κB), and finally leads to the expression of cytokines and costimulatory molecules[5]. Different receptor assemblies, e.g., mono- and heterodimerization, as well as different adapter proteins were identified in this process, and the number of variations and adapter molecules is still increasing[6]. Myeloid differentiation primary response gene 88 (MyD88) is the immediate adapter molecule that is common to all TLRs, except TLR3. In the latter case, Toll/interleukin-1 receptor-like domain (TIR)–containing adapter-inducing interferon-β (TRIF) undertakes this adapter function in order to drive TLR3-dependent gene expression. However, MyD88-independent signaling pathways are also described for many other TLRs. Alternatively, proteins such as TRIF, the TIR domain-containing adapter protein (TIRAP), and TRIF-related adaptor molecule (TRAM) are known to be responsible for signaling transmission downstream of the membrane. In all cases, after ligand binding, the activated membrane receptor complex induces the interleukin-1 receptor-associated kinase (IRAK) and tumor necrosis factor receptor-associated factor (TRAF) family members to facilitate a proper gene expression via transcription factors of the MAPK family, such as the extracellular signal-regulated kinase (ERK)1/2, p38, stress-activated protein kinase/c-Jun N-terminal kinase (SAPK/JNK), and NF-κB[4,5,6].

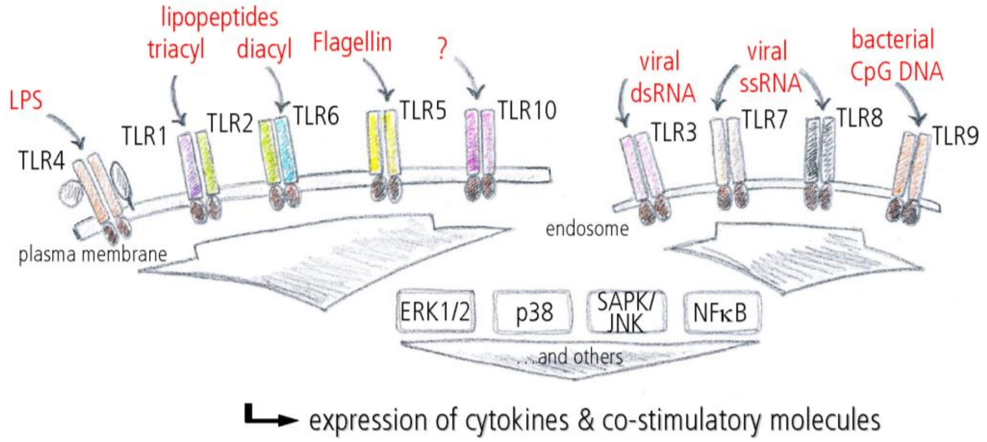

**FIGURE 1.** TLRs and major signaling pathways.

There is accumulating evidence from recent research that TLRs have distinct different functions beyond simple pathogen recognition. In a more complex immunologic view, an important role in dendritic cell maturation and T-cell activation established TLRs as a link between innate and adaptive immunity[7]. Furthermore, the detection of several TLR members in multiple tissues and cell types – besides cells of the immune system – led to a more wide-ranging view on TLRs. Inflammatory disorders such as atherosclerosis[8] and liver disease[9], but also autoimmune diseases[10], are critically influenced by TLRs especially. Moreover, an involvement of TLRs in allograft acceptance/rejection during transplantation[11] or contact allergy to nickel[12] has been shown. Of interest, an interaction of TLRs with endogenous ligands released during tissue damage and fibrosis, or from apoptotic cells, has recently been discovered and seems to regulate many sterile inflammatory processes[13]. These ligands include proteins and peptides, polysaccharides and proteoglycans, nucleic acids, and phospholipids, which are cellular components or extracellular matrix (ECM) degradation products. Accumulating evidence demonstrates that endogenous ligand–mediated TLR signaling is involved in pathological conditions such as tissue injury, autoimmune diseases, and tumorigenesis. The ability of TLRs to recognize endogenous ligands appears to be essential for their function in regulating noninfectious inflammation. Furthermore, a novel role for TLRs in wound healing[14,15] and liver regeneration[16], also in response to endogenous ligands[13], has been reported, suggesting even a regenerative aspect in TLR biology.

## POSTNATAL ANGIOGENESIS – A DOUBLE-EDGED SWORD

Physiological tissue function depends on an adequate supply of nutrients and oxygen through the blood vessels. Consequently, the cardiovascular system is the first organ system that develops during embryogenesis. Angiogenesis is the formation of new blood vessels by sprouting or by intussusception from pre-existing vessels, whereas vasculogenesis characterizes the development of blood vessels by endothelial cell differentiation from progenitor cells; both processes are essential for vascularization during embryonic development[17]. Postnatal vascular network formation is dominated by angiogenesis and vasculogenesis seems to be of minor importance. However, both processes frequently occur in combination and are sometimes subsumed under the more general term neovascularization. The development of the vascular network is a tightly controlled process and is of great physiological as well as pathophysiological relevance. In this regard, postnatal angiogenesis is a double-edged sword; on the one hand, important for wound healing, but on the other hand, detrimental during tumorigenesis[18]. Proliferating endothelial cells initially assemble as solid cords that acquire additional lumen formation. Lumen formation is accomplished by the thinning of endothelial cells or fusion of pre-existing vessels. Further important steps after lumen establishment involve the differentiation of endothelial cells according to environmental demands, maturation into a functional three-dimensional endothelial cell network, and the protection of quiescent endothelial cells from apoptosis[19]. Angiogenesis is initiated and regulated by several growth factors and cytokines. Vascular endothelial growth factor (VEGF) – which was initially described to modify endothelial cell permeability[20] – and its receptor (VEGFR) are a predominant factor and were intensively studied in the last 2 decades[21]. Until now, five VEGF ligands have been identified that occur in different spliced and processed variants, and all of them represent a secreted dimeric glycoprotein of ~40 kDa. In addition to VEGFA–D, placenta growth factor (PLGF) belongs to the VEGF family as well. These ligands bind to the three receptor tyrosine kinases VEGFR1–3 with an overlapping pattern and coreceptors such as heperansulfat, proteoglycans, and neuropilins. Different VEGFRs have distinct different functions; VEGFR1 is involved in the recruitment of hematopoietic progenitor cells and migration of monocytic cells, whereas VEGFR2 and 3 are essential for the function of endothelial cells, especially during angiogenesis.

The Tie receptors, together with their corresponding angiopoietin (Ang) ligands, were identified as the second endothelial cell–specific receptor tyrosine kinase signaling system[22] with important functions for embryonic vascular development as well as for vascular homeostasis in the adult. Ang-Tie signaling controls vessel quiescence and also regulates the later steps of the angiogenic cascade that are related to vessel maturation[23]. Especially during angiogenesis, the interaction of the Ang-Tie system with the VEGF system becomes apparent. Capillaries sprout and subsequently grow alongside a VEGF gradient. Endothelial cells at the leading edge of the migration front, so-called tip cells, exhibit numerous filopodia and express members of the VEGFR family. Subjacent endothelial cells could be subdivided into highly proliferative and differentiating stalk cells and resting phalanx cells, which both express components of the Ang-Tie system[23]. Another key component of sprouting angiogenesis by regulating tip cell vs. stalk cell communication is the highly conserved Delta/Notch signaling system. Mammals possess four different notch receptors, referred to as Notch1–4. Notch receptors are single-pass transmembrane receptors and are capable of binding the membrane-bound ligands Delta-like (Dll) 1–4 and Jagged. Notch signaling in the stalk cells induces a quiescent and nonsprouting phenotype in endothelial cells, whereas adjacent tip cells express Dll4, therefore promoting sprouting activity[24]. Subsequently, factors such as basic fibroblast growth factor (bFGF) and platelet-derived growth factor (PDGF) drive capillary coverage by pericytes and later by smooth muscle cells, which finally guarantees vessel stability and maturation[25]. Taking together, a concerted action of different angiogenic factors mediates capillary sprouting, growth, and subsequent stabilization (Fig. 2). However, many other cytokines

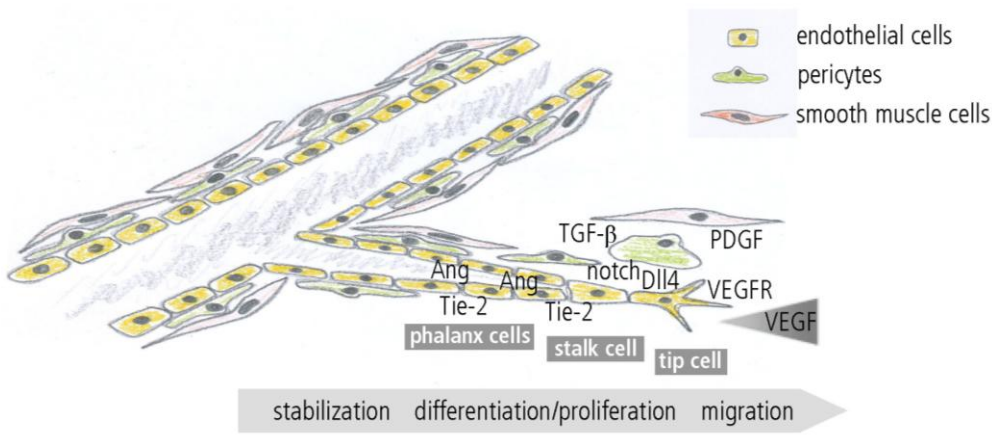

**FIGURE 2.** Postnatal angiogenesis is organized by the concerted action of different angiogenic factors.

and growth factors, including the hematopoietic growth factors granulocyte colony-stimulating factor (G-CSF) and granulocyte-macrophage colony-stimulating factor (GM-CSF), have been shown to exhibit proangiogenic potential[26]. Vice versa, inhibitory factors like angiostatin may prevent the initiation of angiogenesis or keep overwhelming capillary growth in check[25]. Therefore, different pro- and antiangiogenic factors cooperate to regulate the angiogenic process.

Angiogenesis is involved in many physiological and pathophysiological processes, and therefore represents a target of various therapeutic regimes. A proangiogenic concept, for instance, seems to be reasonable for the treatment of patients after myocardial infarction. Infarction leads to myocardial ischemia, followed by destruction of myocardial tissue. Thus, angiogenesis and, consequently, tissue regeneration is a key step for the recovery of cardiac function and, consequently, for the quality of life. In a therapeutic manner, angiogenesis could be significantly improved by the application of growth factors or cytokines, and by the application of stem or progenitor cells, respectively[27]. An opposite therapeutic strategy is pursued for the suppression of tumor growth. The development of solid tumors strictly depends on a growing capillary network – termed tumor angiogenesis – ensuring sufficient supply with oxygen and nutrients. Accordingly, antiangiogenic concepts aim at the inhibition of tumor angiogenesis and thereby tumor nutrient supply[28]. In this regard, the first antitumor therapy with a VEGF-neutralizing monoclonal antibody for the treatment of metastasizing bowel cancer was approved by the U.S. Food and Drug Administration in 2004.

## ANGIOGENESIS IN INFLAMMATORY DISORDERS

Inflammation is not solely a key component of pathogen defense during infection; it also plays a critical role in repair mechanisms, e.g., wound healing and subsequent tissue regeneration. Physiological wound healing requires the integration of complex cellular and molecular events. To take dermal wound healing as an example (Fig. 3), the repair process is tightly controlled, involving different cell types, cytokines, and ECM components during the phases of initial inflammation, as well as the successive cell migration, cell proliferation, and angiogenesis[29]. Of note, cytokines and growth factors released at the site of injury are essential for the repair process[30]. In this regard, angiogenesis, the re-establishment of a capillary network by endothelial cells, is mainly initiated and maintained by the major proangiogenic factor VEGF. Besides endothelial cells, the angiogenic process also involves other cell types, including inflammatory cells, which represent a major source of growth factors and critically contribute to angiogenesis[31]. Platelets, mast cells, but primarily monocytes/macrophages, neutrophils, and other leukocytes

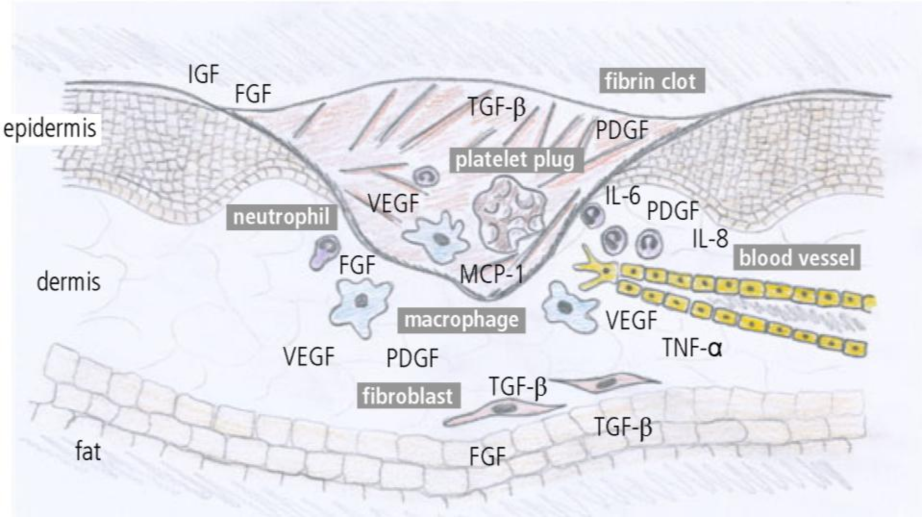

**FIGURE 3.** Dermal wound healing as an example for the interplay of inflammation and angiogenesis.

are recruited to sites of wound healing, partly by the action of the proangiogenic factors such as VEGF. All these cells in turn release proangiogenic factors such as VEGF, bFGF, TGF-β, PDGF, tumor necrosis factor (TNF)-α, insulin-like growth factor (IGF)-1, monocyte chemotactic protein (MCP)-1, interleukin (IL)-6, IL-8, and many more. All these factors finally attract endothelial cells, smooth muscle cells, and fibroblasts to accomplish vessel growth in order to restore sufficient blood supply[32]. Newly formed blood vessels again enhance inflammatory cell recruitment, setting up a stimulating forward loop. In this regard, inflammation often promotes angiogenesis, establishing the term inflammation-induced angiogenesis.

Accumulating evidence suggests an association between angiogenesis and inflammation in pathological situations. Therefore, angiogenesis and inflammation seem to be intimately involved in many chronic inflammatory disorders with distinct etiopathogenic origin, including rheumatoid arthritis, retinopathy, Crohn's disease, diabetes, and cancer. This concept has been further supported by the finding that several previously established noninflammatory disorders, such as obesity, display both inflammation and angiogenesis in an exacerbated manner[33]. In addition, the interplay between inflammatory cells, endothelial cells, and fibroblasts at sites of chronic inflammation, together with the fact that inflammation and angiogenesis can actually be triggered by the same molecular events, further strengthen this association.

## TOLL-LIKE RECEPTORS AT THE INTERFACE OF INFLAMMATION AND ANGIOGENESIS

In general parlance, infection results in inflammation, which in turn promotes angiogenesis, mainly by the action of growth factors released by different leukocytes. Even though the association of inflammation and angiogenesis has been established for quite some time, the knowledge about the role of TLRs in this context is still limited. However, there is a significant number of publications showing that several TLR agonists are able to induce the expression and secretion of angiogenic factors from different cell types *in vitro*. The majority of these studies remains rather descriptive in this context and is very much focused on LPS and VEGF. Up to now, only few data document a direct involvement of TLRs in angiogenesis, both in physiological and in pathophysiological settings.

## Infection and Angiogenesis

Accumulating evidence points to a direct contribution of TLRs to the angiogenic process following bacterial infections, also referred to as infection-induced angiogenesis. In this regard, PAMPs from various bacterial species – superabundant in an infection setting – are known to act via different TLRs. A possible influence of TLRs on angiogenic processes was first discovered in the context of adenosine and its $A_{2A}$ receptor ($A_{2A}$R). The cardioprotective nucleoside adenosine was found to stimulate angiogenesis through up-regulation of VEGF, thereby participating in tissue protection following ischemic events. In 2002, Leibovich et al. described a synergistic interaction of $A_{2A}$R agonists with LPS through the TLR4 pathway. This interaction resulted in a strong up-regulation of VEGF and down-regulation of TNF-α in macrophages[34], and could also be demonstrated for TLR2, 7, and 9[35], representing an angiogenic switch. This synergy observed *in vitro* also seems to play an important role *in vivo*, given the fact that MyD88-deficient mice showed markedly slower wound healing and reduced generation of new capillaries in response to an $A_{2A}$R agonist[36]. In terms of TLR4, it is very likely that LPS induces adenosine, which in turn promotes angiogenesis through $A_{2A}$R by the up-regulation of VEGF expression in macrophages[37].

Independent of the $A_{2A}$R system, Pollet et al. showed that the TLR4 ligand LPS directly stimulates endothelial sprouting *in vitro* via a TRAF6-, NF-κB–, and JNK-dependent mechanism. However, the responsible angiogenic growth factor remained elusive in this context[38]. Furthermore, a so-far-unidentified TLR ligand seems to be involved in the formation of angiogenic lesions resulting from infection with the facultative intracellular bacterium *Bartonella henselae*. This bacterial infection leads to enhanced MCP-1 production in endothelial cells, which in turn induces chemotaxis of monocytes in order to initiate angiogenesis by VEGF production. Interestingly, MCP-1 production was independent of LPS/TLR4, but dependent on NF-κB[39]. A serious problem of severe ocular infection is pathological corneal neovascularization, which could finally lead to impaired eyesight. In this regard, it has been shown that VEGF and TLR4 expression are up-regulated in response to LPS and that VEGF expression is TLR4 dependent[40].

However, angiogenesis also contributes to the regeneration process during liver fibrosis, which is associated with increased endotoxin levels in the gut and portal circulation. Jagavelu and colleagues recently demonstrated a key role for the TLR4/MyD88 axis during VEGF production and the subsequent angiogenic process in liver endothelial cells following LPS stimulation[41]. Likewise, mycoplasma infections could be accompanied by enhanced angiogenesis and microvascular remodeling, which are features of chronic inflammation as elicited by *Mycoplasma pulmonis* infections of the respiratory tract[42]. In this regard, we recently investigated the highly angiogenic properties of the specific TLR2/6 agonist macrophage-activating lipopeptide of 2 kDa (MALP-2), a diacylated lipopeptide that occurs in mycoplasma species and Gram-positive bacteria. Interestingly, this process seems to be independent of VEGF. We discovered a TLR2/6-dependent induction of the MAPK cascade and NF-κB, and strong secretion of GM-CSF, in particular from endothelial cells and to a lesser degree from monocytes. Accordingly, MALP-2–induced angiogenesis *in vitro* and *in vivo* could be suppressed by inhibition of GM-CSF[43].

In addition to an acute infection upon injury, there are different acute or chronic inflammatory disorders that are also associated with bacterial infection independent of an initial injury. Arthritis is characterized by inflammatory cell infiltration into the concerned joint. Progression of the disease includes self-perpetuating destruction of articular cartilage and extensive angiogenesis in the synovial membrane. TLR2 ligands of Gram-positive bacteria, such as peptidoglycan (PGN), seem especially to be responsible for this angiogenic phenotype characterized by the induction of VEGF in chondrocytes[44] and, accordingly, VEGF and IL-8 in fibroblasts[45].

## Tumor Angiogenesis

The role of TLRs in tumor angiogenesis is quite diverse just as cancer is itself. Cyclooxygenase-2 (COX-2) is known to play a crucial role in *Helicobacter pylori*–associated gastric cancer. In this regard, Chang and coworkers demonstrated that *H. pylori* acts through TLR2 and 9 to activate the MAPK cascade, leading to COX-2–dependent prostaglandin $E_2$ ($PGE_2$) release and thereby contributing to cancer cell invasion and angiogenesis[46]. On the other hand, stimulation of TLRs with particular agonists can also cause antitumor activity, interfering with cancer proliferation and angiogenesis by a mechanism still incompletely understood. For instance, the immunomodulatory TLR9 agonist IMO inhibited microvessel formation and tumor growth[47]. Especially, chronic lymphocytic leukemia seemed to be amenable to TLR agonists. In all likelihood, there are two different possibilities for TLR agonists to limit tumor growth: first, by altering the tumor microenvironment and inhibiting angiogenesis, and second, by clearing tumor cells due to enhanced activity of natural-killer and tumor-reactive T cells. In this regard, the TLR7 agonist imidazoquinoline and the TLR9 agonist unmethylated cytosine-phosphate-guanosine (CpG) oligonucleotide were shown to exhibit strong local activity against leukemia, and respective phase I trials are currently in progress at different centers[48].

## Endogenous Toll-Like Receptor Ligands and Angiogenesis

Sustained proinflammatory responses in diseases such as rheumatoid arthritis, atherosclerosis, diabetic retinopathy, and cancer are often associated with increased angiogenesis that contributes to tissue disruption and disease progression. In this context, the high-mobility group B1 (HMGB1), which is released by necrotic cells, has been recognized to signal through the receptor for advanced glycation end products (RAGE), and via TLR2 and TRL4. Activation of these receptors resulted in the activation of NF-κB and the up-regulation of angiogenic factors like VEGF in both hematopoietic and endothelial cells[49]. Following this line, inflammation-induced oxidative stress and angiogenesis is emerging as an important mechanism underlying numerous processes, from tissue regeneration and remodeling to cancer progression. Interestingly, West and coworkers recently reported that end products of lipid oxidation such as ω-(2-carboxyethyl)pyrrole (CEP) are generated and accumulate during inflammation, wound healing, and in tumors. CEP is specifically recognized by TLR2, but not TLR4 or scavenger receptors in endothelial cells, leading to a MyD88-dependent angiogenic response that is independent of VEGF[50]. Apparently, endogenous ligands, which accumulate during inflammatory tissue disruption and enhanced oxidative stress conditions, are also capable of promoting angiogenesis via a TLR-dependent pathway.

## SUMMARY AND PERSPECTIVE

Accumulating evidence points to a crucial role of TLRs in angiogenesis. However, the mode of action of TLRs in this context is quite diverse. TLR activation consistently promotes angiogenesis in various inflammatory settings in response to both exogenous and endogenous ligands. In regard to an acute local infectious scenario, the angiogenic process seems to be important for sufficient blood supply, and the recruitment of immune competent cells for pathogen clearance and subsequent tissue regeneration. In contrast, chronic local infection or prolonged pathogen-independent inflammation leads to excessive angiogenesis with eventual pathological consequences. The role of TLRs in tumor angiogenesis is even more contradictory since pro- as well as antiangiogenic properties are reported. Fig. 4 illustrates a snap-shot of the current understanding of the involvement of TLRs in angiogenesis.

Of note, everything discussed in this review deals with postnatal angiogenesis, whereas the role of TLRs in angiogenesis during embryonic development is completely unknown. Hence, more basic knowledge is needed to fully understand the role of different TLRs in angiogenesis.

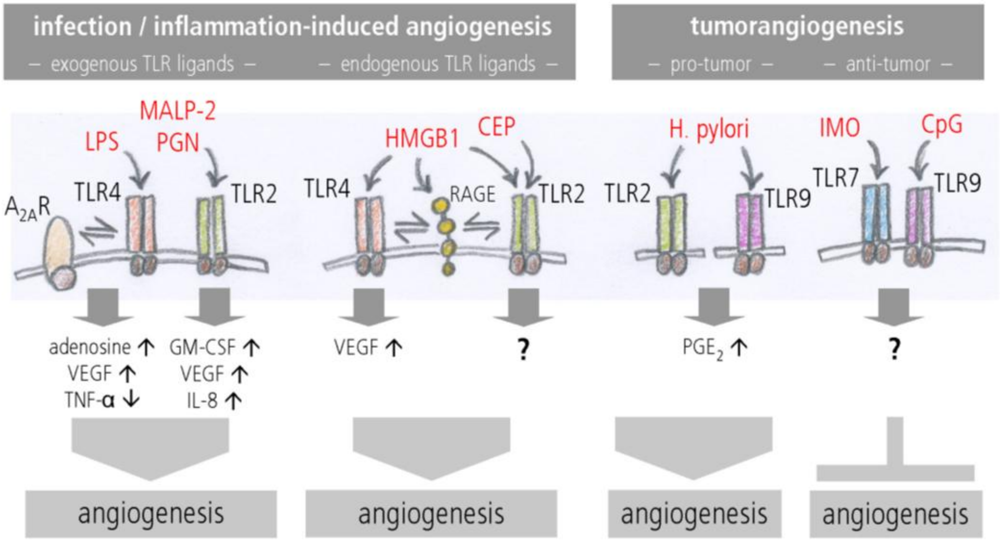

**FIGURE 4.** Current understanding of the involvement of TLRs in angiogenesis.

In the future, modulation of TLR signaling could provide the basis for the development of novel therapeutic approaches in diverse settings. From this point of view, inhibitory strategies targeting TLR signaling seem to be plausible in chronic and persistent infectious situations such as rheumatoid arthritis. Small molecules or siRNA against specific TLRs or their downstream targets may provide novel tools to combat local inflammation via inhibition of angiogenesis. Especially, advanced tissue penetration properties of those engineered molecules render them applicable and superior for the use in tissues that are inaccessible for antibiotics. Likewise, inhibitory strategies targeting TLRs could be used to inhibit pathological tumor angiogenesis in order to limit tumor growth. In particular, modulation of TLR7 and 9 activity seems to be a potential future therapeutic target[46,47,48]. However, great caution is required since pro- as well as antiangiogenic properties with subsequent pro- or antitumorigenic properties of different agonists recognized by the same TLR are reported[46,47].

In our opinion, the most promising future therapeutic approach is the application of specific TLR agonists in damaged ischemic or hypoxic tissues in order to promote angiogenesis and subsequent tissue regeneration, especially when the tissue damage is not initiated or accompanied by severe infection. In such settings, a single application of TLR agonists mimics an infectious scenario without prolonged local pathogen presences. Such an initial therapeutic boost of the immune system with a specific TLR agonist aims to launch a defined regenerative program, including enhanced angiogenesis. Of note, the application of single proangiogenic growth factors has already been tested in clinical trials. However, in the case of VEGF monotherapy, large-scale trials have not yet yielded consistent beneficial results[51,52]. This may be related to recent observations that several other potent proangiogenic factors act in concert with VEGF for proper vessel formation and maturation[23,24,25]. Therefore, stimulation of specific TLRs may provide an opportunity to induce a specific pattern of proangiogenic growth factors for sufficient tissue regeneration. In this regard, we pin our hope on naturally occurring substances such as the lipopeptide and TLR2/6 agonist MALP-2. As already mentioned, we recently reported that the proangiogenic properties of MALP-2 critically depended on the induction of the growth factor GM-CSF in endothelial cells and monocytes[43]. Moreover, yet-unpublished observations of our group showed even promising effects of MALP-2 on endothelial regeneration after vascular injury. Those experimental data render MALP-2 eligible for the future treatment of patients, e.g., with diabetes, who suffer from peripheral vascular damage with insufficient angiogenesis; ideally, locally applied with a drug-eluting stent, widely used in interventional cardiovascular medicine.

Taken together, modulation of TLR activity could be used for different future therapeutic concepts; on the one hand, to inhibit pathological tumor angiogenesis to limit tumor growth and on the other hand, to promote physiological angiogenesis for tissue regeneration.

## ACKNOWLEDGMENTS

The work of KG and BS is supported by grants from the German Research Foundation (DFG) KFO 136 and SFB 566/b9, and from the Federal Ministry of Education and Research (BMBF) 01GU0711.

## References

1. Taguchi, T., Mitcham, J.L., Dower, S.K., Sims, J.E., and Testa, J.R. (1996) Chromosomal localization of TIL, a gene encoding a protein related to the Drosophila transmembrane receptor Toll, to human chromosome 4p14. *Genomics* **32,** 486–488.
2. Medzhitov, R., Preston-Hurlburt, P., and Janeway, C.A., Jr. (1997) A human homologue of the Drosophila Toll protein signals activation of adaptive immunity. *Nature* **388,** 394–397.
3. Poltorak, A., He, X., Smirnova, I., Liu, M.Y., Van Huffel, C., Du, X., Birdwell, D., Alejos, E., Silva, M., Galanos, C., Freudenberg, M., Ricciardi-Castagnoli, P., Layton, B., and Beutler, B. (1998) Defective LPS signaling in C3H/HeJ and C57BL/10ScCr mice: mutations in Tlr4 gene. *Science* **282,** 2085–2088.
4. Takeda, K., Kaisho, T., and Akira, S. (2003) Toll-like receptors. *Annu. Rev. Immunol*. **21,** 335–376.
5. Oda, K. and Kitano, H. (2006) A comprehensive map of the toll-like receptor signaling network. *Mol. Syst. Biol*. **2,** 2006.0015.
6. Brikos, C. and O'Neill, L.A. (2008) Signalling of toll-like receptors. *Handb. Exp. Pharmacol*. 21–50.
7. Kaisho, T. and Akira, S. (2003) Regulation of dendritic cell function through toll-like receptors. *Curr. Mol. Med*. **3,** 759–771.
8. Satoh, M., Ishikawa, Y., Minami, Y., Takahashi, Y., and Nakamura, M. (2008) Role of Toll like receptor signaling pathway in ischemic coronary artery disease. *Front. Biosci*. **13,** 6708–6715.
9. Seki, E. and Brenner, D.A. (2008) Toll-like receptors and adaptor molecules in liver disease: update. *Hepatology* **48,** 322–335.
10. Marshak-Rothstein, A. (2006) Toll-like receptors in systemic autoimmune disease. *Nat. Rev. Immunol*. **6,** 823–835.
11. Obhrai, J. and Goldstein, D.R. (2006) The role of toll-like receptors in solid organ transplantation. *Transplantation* **81,** 497–502.
12. Schmidt, M., Raghavan, B., Müller, V., Vogl, T., Fejer, G., Tchaptchet, S., Keck, S., Kalis, C., Nielsen, P.J., Galanos, C., Roth, J., Skerra, A., Martin, S.F., Freudenberg, M.A., Goebeler, M. (2010) Crucial role for human Toll-like receptor 4 in the development of contact allergy to nickel. *Nat. Immunol*. **11,** 814–819.
13. Yu, L., Wang, L., and Chen, S. (2010) Endogenous Toll-like receptor ligands and their biological significance. *J. Cell. Mol. Med*. **14,** 2592–2603
14. Deiters, U., Barsig, J., Tawil, B., and Muhlradt, P.F. (2004) The macrophage-activating lipopeptide-2 accelerates wound healing in diabetic mice. *Exp. Dermatol*. **13,** 731–739.
15. Macedo, L., Pinhal-Enfield, G., Alshits, V., Elson, G., Cronstein, B.N., and Leibovich, S.J. (2007) Wound healing is impaired in MyD88-deficient mice: a role for MyD88 in the regulation of wound healing by adenosine A2A receptors. *Am. J. Pathol*. **171,** 1774–1788.
16. Seki, E., Tsutsui, H., Iimuro, Y., Naka, T., Son, G., Akira, S., Kishimoto, T., Nakanishi, K., and Fujimoto, J. (2005) Contribution of Toll-like receptor/myeloid differentiation factor 88 signaling to murine liver regeneration. *Hepatology* **41,** 443–450.
17. Ribatti, D. (2010) The seminal work of Werner Risau in the study of the development of the vascular system. *Int. J. Dev. Biol*. **54,** 567–572.
18. Pandya, N.M., Dhalla, N.S., and Santani, D.D. (2006) Angiogenesis--a new target for future therapy. *Vascul. Pharmacol*. **44,** 265–274.
19. Senger, D.R., Galli, S.J., Dvorak, A.M., Perruzzi, C.A., Harvey, V.S., and Dvorak, H.F. (1983) Tumor cells secrete a vascular permeability factor that promotes accumulation of ascites fluid. *Science* **219,** 983–985.
20. Carmeliet, P. (2000) Mechanisms of angiogenesis and arteriogenesis. *Nat. Med*. **6,** 389–395.
21. Olsson, A.K., Dimberg, A., Kreuger, J., and Claesson-Welsh, L. (2006) VEGF receptor signalling - in control of vascular function. *Nat. Rev. Mol. Cell Biol*. **7,** 359–371.
22. Partanen, J., Armstrong, E., Mäkelä, T.P., Korhonen, J., Sandberg, M., Renkonen, R., Knuutila, S., Huebner, K., and Alitalo, K. (1992) A novel endothelial cell surface receptor tyrosine kinase with extracellular epidermal growth factor homology domains. *Mol. Cell. Biol*. **12,** 1698–1707.

23. Augustin, H.G., Koh, G.Y., Thurston, G., and Alitalo, K. (2009) Control of vascular morphogenesis and homeostasis through the angiopoietin-Tie system. *Nat. Rev. Mol. Cell Biol*. **10,** 165–177.
24. Gridley, T. (2010) Notch signaling in the vasculature. *Curr. Top. Dev. Biol*. **92,** 277–309.
25. Distler, J.H., Hirth, A., Kurowska-Stolarska, M., Gay, R.E., Gay, S., and Distler, O. (2003) Angiogenic and angiostatic factors in the molecular control of angiogenesis. *Q. J. Nucl. Med*. **47,** 149–161.
26. Bussolino, F., Ziche, M., Wang, J.M., Alessi, D., Morbidelli, L., Cremona, O., Bosia, A., Marchisio, P.C., and Mantovani, A. (1991) In vitro and in vivo activation of endothelial cells by colony-stimulating factors. *J. Clin. Invest*. **87,** 986–995.
27. Vandervelde, S., van Luyn, M.J., Tio, R.A., and Harmsen, M.C. (2005) Signaling factors in stem cell-mediated repair of infarcted myocardium. *J. Mol. Cell. Cardiol*. **39,** 363–376.
28. Cao, Y. (2009) Tumor angiogenesis and molecular targets for therapy. *Front. Biosci*. **14,** 3962–3973.
29. Yamaguchi, Y. and Yoshikawa, K. (2001) Cutaneous wound healing: an update. *J. Dermatol*. **28,** 521–534.
30. Gharaee-Kermani, M. and Phan, S.H. (2001) Role of cytokines and cytokine therapy in wound healing and fibrotic diseases. *Curr. Pharm. Des*. **7,** 1083–1103.
31. Frantz, S., Vincent, K.A., Feron, O., and Kelly, R.A. (2005) Innate immunity and angiogenesis. *Circ. Res*. **96,** 15–26.
32. Carmeliet, P. (2005) Angiogenesis in life, disease and medicine. *Nature* **438,** 932–936.
33. Costa, C., Incio, J., and Soares, R. (2007) Angiogenesis and chronic inflammation: cause or consequence? *Angiogenesis* **10,** 149–166.
34. Leibovich, S.J., Chen, J.F., Pinhal-Enfield, G., Belem, P.C., Elson, G., Rosania, A., Ramanathan, M., Montesinos, C., Jacobson, M., Schwarzschild, M.A., Fink, J.S., and Cronstein, B. (2002) Synergistic up-regulation of vascular endothelial growth factor expression in murine macrophages by adenosine A(2A) receptor agonists and endotoxin. *Am. J. Pathol*. **160,** 2231–2244.
35. Pinhal-Enfield, G., Ramanathan, M., Hasko, G., Vogel, S.N., Salzman, A.L., Boons, G.J., and Leibovich, S.J. (2003) An angiogenic switch in macrophages involving synergy between Toll-like receptors 2, 4, 7, and 9 and adenosine A(2A) receptors. *Am. J. Pathol*. **163,** 711–721.
36. Macedo, L., Pinhal-Enfield, G., Alshits, V., Elson, G., Cronstein, B.N., and Leibovich, S.J. (2007) Wound healing is impaired in MyD88-deficient mice: a role for MyD88 in the regulation of wound healing by adenosine A2A receptors. *Am. J. Pathol*. **171,** 1774–1788.
37. Hara, Y., Kuroda, N., Inoue, K., and Sato, T. (2009) Up-regulation of vascular endothelial growth factor expression by adenosine through adenosine A2 receptors in the rat tongue treated with endotoxin. *Arch. Oral Biol*. **54,** 932–942.
38. Pollet, I., Opina, C.J., Zimmerman, C., Leong, K.G., Wong, F., and Karsan, A. (2003) Bacterial lipopolysaccharide directly induces angiogenesis through TRAF6-mediated activation of NF-kappaB and c-Jun N-terminal kinase. *Blood* **102,** 1740–1742.
39. McCord, A.M., Burgess, A.W., Whaley, M.J., and Anderson, B.E. (2005) Interaction of Bartonella henselae with endothelial cells promotes monocyte/macrophage chemoattractant protein 1 gene expression and protein production and triggers monocyte migration. *Infect. Immun*. **73,** 5735–5742.
40. Rodríguez-Martínez, S., Cancino-Diaz, M.E., Miguel, P.S., and Cancino-Diaz, J.C. (2006) Lipopolysaccharide from Escherichia coli induces the expression of vascular endothelial growth factor via toll-like receptor 4 in human limbal fibroblasts. *Exp. Eye Res*. **83;** 1373–1377.
41. Jagavelu, K., Routray, C., Shergill, U., O'Hara, S.P., Faubion, W., and Shah, V.H. (2010) Endothelial cell toll-like receptor 4 regulates fibrosis-associated angiogenesis in the liver. *Hepatology* **52,** 590–601.
42. McDonald, D.M. (2001) Angiogenesis and remodeling of airway vasculature in chronic inflammation. *Am. J. Respir. Crit. Care Med*. **164,** 39–45.
43. Grote, K., Schuett, H., Salguero, G., Grothusen, C., Jagielska, J., Drexler, H., Mühlradt, P.F., and Schieffer, B. (2010) Toll-like receptor 2/6 stimulation promotes angiogenesis via GM-CSF as a potential strategy for immune defense and tissue regeneration. *Blood* **115**, 2543–2552.
44. Varoga, D., Paulsen, F., Mentlein, R., Fay, J., Kurz, B., Schütz, R., Wruck, C., Goldring, M.B., and Pufe, T. (2006) TLR-2-mediated induction of vascular endothelial growth factor (VEGF) in cartilage in septic joint disease. *J. Pathol*. **210,** 315–324.
45. Cho, M.L., Ju, J.H., Kim, H.R., Oh, H.J., Kang, C.M., Jhun, J.Y., Lee, S.Y., Park, M.K., Min, J.K., Park, S.H., Lee, S.H., and Kim, H.Y. (2007) Toll-like receptor 2 ligand mediates the upregulation of angiogenic factor, vascular endothelial growth factor and interleukin-8/CXCL8 in human rheumatoid synovial fibroblasts. *Immunol. Lett*. **108,** 121–128.
46. Chang, Y.J., Wu, M.S., Lin, J.T., and Chen, C.C. (2005) Helicobacter pylori-induced invasion and angiogenesis of gastric cells is mediated by cyclooxygenase-2 induction through TLR2/TLR9 and promoter regulation. *J. Immunol*. **175,** 8242–8252.
47. Damiano, V., Caputo, R., Bianco, R., D'Armiento, F.P., Leonardi, A., De Placido, S., Bianco A.R., Agrawal, S., Ciardiello, F., and Tortora, G. (2006) Novel toll-like receptor 9 agonist induces epidermal growth factor receptor (EGFR) inhibition and synergistic antitumor activity with EGFR inhibitors. *Clin. Cancer Res*. **12,** 577–583.
48. Spaner, D.E. and Masellis, A. (2007) Toll-like receptor agonists in the treatment of chronic lymphocytic leukemia. *Leukemia* **21,** 53–60.

49. van Beijnum, J.R., Buurman, W.A., and Griffioen, A.W. (2008) Convergence and amplification of toll-like receptor (TLR) and receptor for advanced glycation end products (RAGE) signaling pathways via high mobility group B1 (HMGB1). *Angiogenesis* **11,** 91–99.
50. West, X.Z., Malinin, N.L., Merkulova, A.A., Tischenko, M., Kerr, B.A., Borden, E.C., Podrez, E.A., Salomon, R.G., and Byzova, T.V. (2010) Oxidative stress induces angiogenesis by activating TLR2 with novel endogenous ligands. *Nature* **467,** 972–976.
51. Freedman, S.B., Vale, P., Kalka, C., Kearney, M., Pieczek, A., Symes, J., Losordo, D., and Isner, J.M. (2002) Plasma vascular endothelial growth factor (VEGF) levels after intramuscular and intramyocardial gene transfer of VEGF-1 plasmid DNA. *Hum. Gene Ther*. **13**, 1595–1603.
52. Henry, T.D., Annex, B.H., McKendall, G.R., Azrin, M.A., Lopez, J.J., Giordano, F.J., Shah, P.K., Willerson, J.T., Benza, R.L., Berman, D.S., Gibson, C.M., Bajamonde, A., Rundle, A.C., Fine, J., McCluskey, E.R., and VIVA Investigators (2003) The VIVA trial: Vascular endothelial growth factor in Ischemia for Vascular Angiogenesis. *Circulation* **107**, 1359–1365.
